# Evaluating the stability of synthetic cathinones in liquid urine and dried urine spots: impact of pH and storage conditions

**DOI:** 10.1007/s00204-025-04272-0

**Published:** 2025-12-21

**Authors:** Stefania Boccuzzi, David Cowan, Paul I. Dargan, Edward Goucher, Vincenzo Abbate

**Affiliations:** 1 Department of Analytical, Environmental & Forensic Sciences, Faculty of Life Sciences & Medicine, King’s College London, London, UK; 2 Clinical Toxicology, Faculty of Life Sciences and Medicine, King’s College London, London, UK; 3 Clinical Toxicology, Guy’s and St Thomas’ NHS Foundation Trust and King’s Health Partners, London, UK; 4 Thermo Fisher Scientific, San Jose, CA USA

**Keywords:** Synthetic cathinones, Dried matrix spots, Ambient ionisation, Paper spray ionisation, Analyte stability, Urinary pH

## Abstract

Synthetic cathinones comprise a class of new psychoactive substances associated with significant morbidity and mortality. Their detection in biological samples is complicated by their instability, which can compromise quantification or interpretation. This study evaluates the stability of five synthetic cathinones – 4-chloroethcathinone (4-CEC), 4-ethylmethcathinone (4-EMC), N-ethylhexedrone (NEH), methylenedioxypyrovalerone (MDPV) and 4-chloro-α-pyrrolidinopropiophenone (4-Cl-α-PPP) – in dried urine spots (DUS) and liquid urine at acidic (pH 5.98) and mildly basic (pH 7.63) conditions. Fortified samples were stored at room temperature (25 °C) and refrigerated (4 °C) for up to 14 days and analysed using the Thermo Fisher VeriSpray™ Paper Spray Ion Source coupled to a Thermo Fisher Altis™ Plus Triple Quadrupole Mass Spectrometer. Urinary pH was the critical determinant of stability. Under acidic conditions, 4-EMC, 4-Cl-α-PPP, and MDPV retained > 90% of their initial concentration by day 14 in both matrices. Conversely, under basic conditions, 4-CEC and NEH degraded rapidly at room temperature (< 20% remaining by day three). Refrigeration slowed but did not prevent degradation. DUS offered a minor stabilising effect for most analytes, although NEH exhibited greater degradation. Statistically significant differences between acidic and basic conditions were observed (*p* < 0.0083), while matrix format had limited influence at acidic pH. Overall, pH rather than matrix type was the main driver of synthetic cathinone stability in urine. Nevertheless, DUS offer practical benefits for sample transport and storage in forensic settings. Future research should further characterise the physicochemical mechanisms underlying synthetic cathinone degradation in urine to inform the interpretation of operational toxicology workflows.

**Supplementary Information:**

The online version contains supplementary material available at 10.1007/s00204-025-04272-0.

## Introduction

**Fig. 1:**
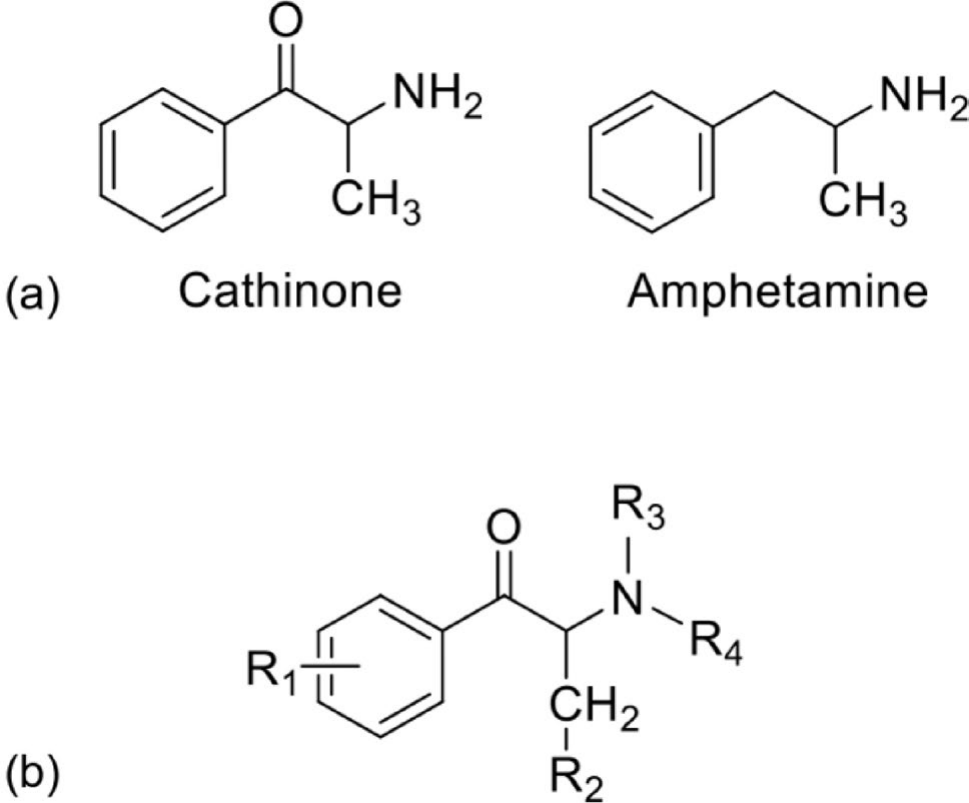
The chemical structure of cathinone compared to that of amphetamine, displaying the addition of a β-ketone on the amino alkyl chain (**a**) and the generic structure of synthetic cathinone analogues; common constituents are shown by R_1_ to R_4_ (**b**)

Synthetic cathinones comprise a class of new psychoactive substances (NPS), with reports of synthetic derivatives being manufactured as early as the 1920s. Recreational synthetic cathinone abuse did not become pronounced until the mid-2000s with the emergence of mephedrone (Wood and Dargan 2012; Dargan et al. 2011). The increase in availability and use of synthetic cathinones is thought to have been related to increased costs and decreased purity of traditional controlled stimulants such as cocaine and 3,4-methylenedioxymethamphetamine (MDMA) (Prosser and Nelson 2012) as well as colloquially being known as “legal highs”, often occupying legally ambiguous status depending on the chemical structure of the modified analogue (Coppola and Mondola 2012). Cathinone itself, originally isolated from the *Catha edulis* plant, is a monoamine alkaloid with structural similarity to amphetamine, differing by the presence of a β-ketone (-oxo) group on the amino alkyl chain (Fig. 1a) (Logan et al. 2017; Valente et al. 2014). Additional psychoactive alkaloids of *Catha edulis* are D-norpseudoephedrine (cathine), with a hydroxyl group replacing the -oxo group, and norephedrine, which lacks β-keto substitution but carries a hydroxyl group (Kalix 1990). The majority of synthetic cathinone analogues are synthesised by the addition of various substituents across the cathinone molecule (Fig. 1b) (Valente et al. 2014), which influence blood–brain barrier penetration and stimulant activity.

Synthetic cathinones exert their stimulant effects through norepinephrine, dopamine, and serotonin transporters by increasing synaptic concentrations of these neurotransmitters. However, the overall pharmacodynamic and pharmacokinetic profiles of synthetic cathinones vary between and within their structural classifications (Schifano et al. 2020). Rodent studies have demonstrated the dependence capability of synthetic cathinones, highlighting the potential impact of compulsive or chronic use (Watterson et al. 2012, 2014; Baumann et al. 2014). Use of synthetic cathinones is associated with significant acute adverse health effects related to their stimulant effects (Hill and Dargan 2018). Synthetic cathinones are the second largest group of NPS being monitored by the European Early Warning System at the European Union Drugs Agency (EUDA) with 178 synthetic cathinone analogues reported to date at the time of publication (EUDA 2025a). Recent trends have seen a decrease in the number of new synthetic cathinone analogues emerging on the European market, from 31 substances in 2014 to just 7 in 2024; however there is persistence of synthetic cathinones on the European market with over 60 previously reported synthetic cathinones detected in 2023 (EUDA 2025b). Further, the quantity of synthetic cathinones seized in Europe has increased since 2020, with 26.5 tonnes of synthetic cathinones seized in 2022, compared with just 4.5 tonnes the year before (EUDA 2024), highlighting that synthetic cathinone availability remains a prevalent and consistent issue in the European market since their inception over two decades ago.

In light of the widespread availability and harms associated with the use of synthetic cathinones, it is important to ensure that analytical techniques provide reliable data. The stability of analytes between collection and analysis can influence the accuracy of drug detection and quantification in biological samples. Analyte stability can be dependent on a variety of factors, ranging from chemical or matrix degradation and enzymatic metabolism, to unpredictable setbacks such as instrument malfunction, or improper transportation or handling of a sample (Jiménez et al. 2006). As such, it is essential to understand the stability of analytes under common storage conditions to predict degradation patterns. Extensive stability studies of synthetic cathinones have been published in recent years, indicating that the majority of synthetic cathinones are unstable under the common storage conditions encountered in forensic toxicology. Outside of predictable influences such as the storage temperature, factors impacting synthetic cathinone instability include the pH of the sample, with acidic conditions exerting a protective effect against degradation, and structural modifications such as halogenated constituents around the cathinone core (Aldubayyan et al. 2021). A lesser discussed factor influencing synthetic cathinone stability is the state of the matrix itself. Matrices containing common drugs of abuse have been shown to be more stable when stored as dried matrix spots (DMS), believed to be due to sample dehydration, minimising enzymatic and chemical hydrolysis processes (Jacques et al. 2022). However, this stabilising effect is highly analyte-dependent, even between drug classes, with some analytes exhibiting minimal improvement (Rossi et al. 2025; Ambach et al. 2014). The use of DMS also promotes easier sample collection with smaller matrix volume, which in turn facilitate advantageous sample handling, transport, and storage; however, it does require extensive sample considerations such as possible heterogeneity in analyte distribution and varied extraction efficiency during subsequent extraction of the analyte from DMS and paper substrate (Jacques et al. 2022; Stove et al. 2012). These processes are often time-consuming and labour-intensive. Instead, if DMS are analysed directly by ambient ionisation techniques, analytical sample preparation and extraction processes are eliminated; from a stability perspective, this would further complement the time-sensitive nature of analysing a sample that has already been stored for longer than preferred.

**Fig. 2:**
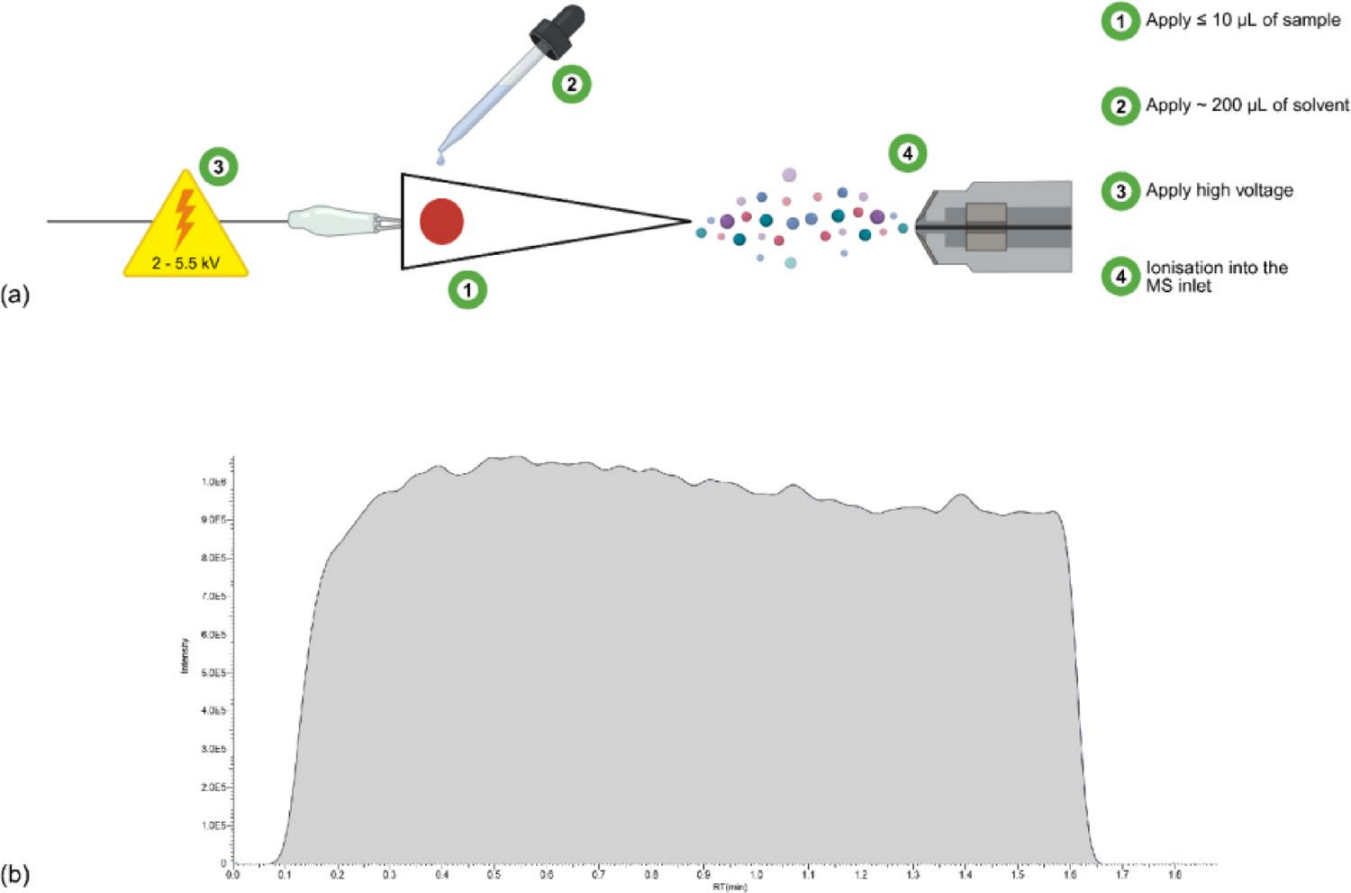
Schematic of the PSI mechanism for the analysis of a biological matrix spot (**a**) and the resulting data output in the form of a representative chronogram (**b**). Figure 2a was generated using BioRender

**Fig. 3:**
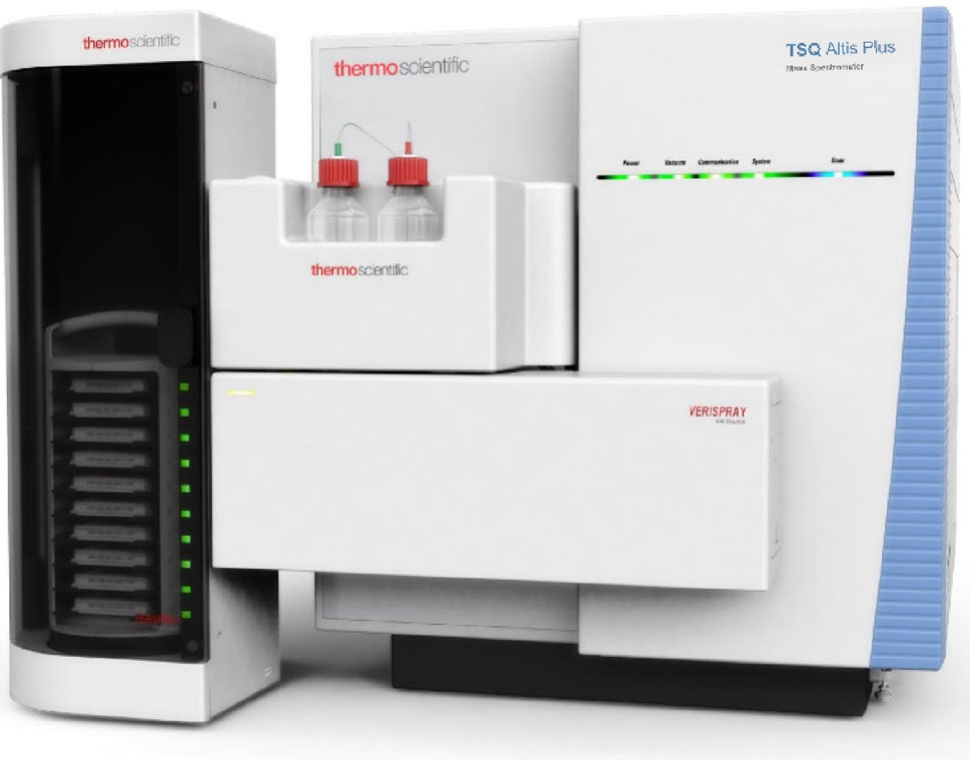
The VeriSpray Paper Spray Ion Source coupled to an Altis Plus Mass Spectrometer. For automation, the VeriSpray source is equipped with a plate magazine and a plate loader, housing up to 10 sample plates each with 24 paper strips embedded enabling unattended runs. For Research Use Only

Ambient ionisation mass spectrometry (AIMS) enables ionisation to occur directly from the sample surface at atmospheric pressure and temperature, promoting simultaneous ionisation and analyte desorption from the substrate. Based on electrospray ionisation (ESI) mechanisms, paper spray ionisation (PSI) was first introduced as an AIMS technique by the Cooks team in 2010 (Wang et al. 2010). Broadly, a small volume of matrix (up to 10 μL) is pipetted onto a paper strip cut into a triangular shape and left to dry. The tip of the strip is positioned in close proximity to a mass spectrometer inlet, and solvents are applied that wick through the paper by capillary action, serving a dual purpose of wetting the paper and extracting the analytes from the matrix and paper substrate. A high voltage is applied (3–5 kV) at the back of the paper strip, which promotes the formation of electrospray at the tip of the paper (Fig. 2a). Data output is in the form of a so called chronogram, displaying a total response of the analyte as a broad curve (Fig. 2b) (Shi et al. 2022). The VeriSpray Paper Spray Ionisation Source coupled to an Altis Plus Triple Quadrupole Mass Spectrometer automates the ionisation process once the matrix spot has dried, enabling fast, unassisted analysis with run times of approximately two minutes per sample (Fig. 3). Compared with traditional LC–MS techniques, PSI omits the chromatographic interface, resulting in fewer consumables such as LC columns, mobile phases, and extraction materials. The combination of high analytical throughput and minimal sample handling makes this approach well suited to rapid toxicological screening, including emergency triage, or point-of-collection DMS analysis, and preliminary anti-doping assessments before confirmatory LC–MS testing. From a stability perspective, direct sample analysis omits the need for sample extraction from the matrix spot, as per traditional DMS extraction, if the sample were to be analysed by chromatographic techniques such as LC–MS. These features further complement the simplicity of the use of DMS for improved stability and faster, more efficient analysis.

**Fig. 4:**
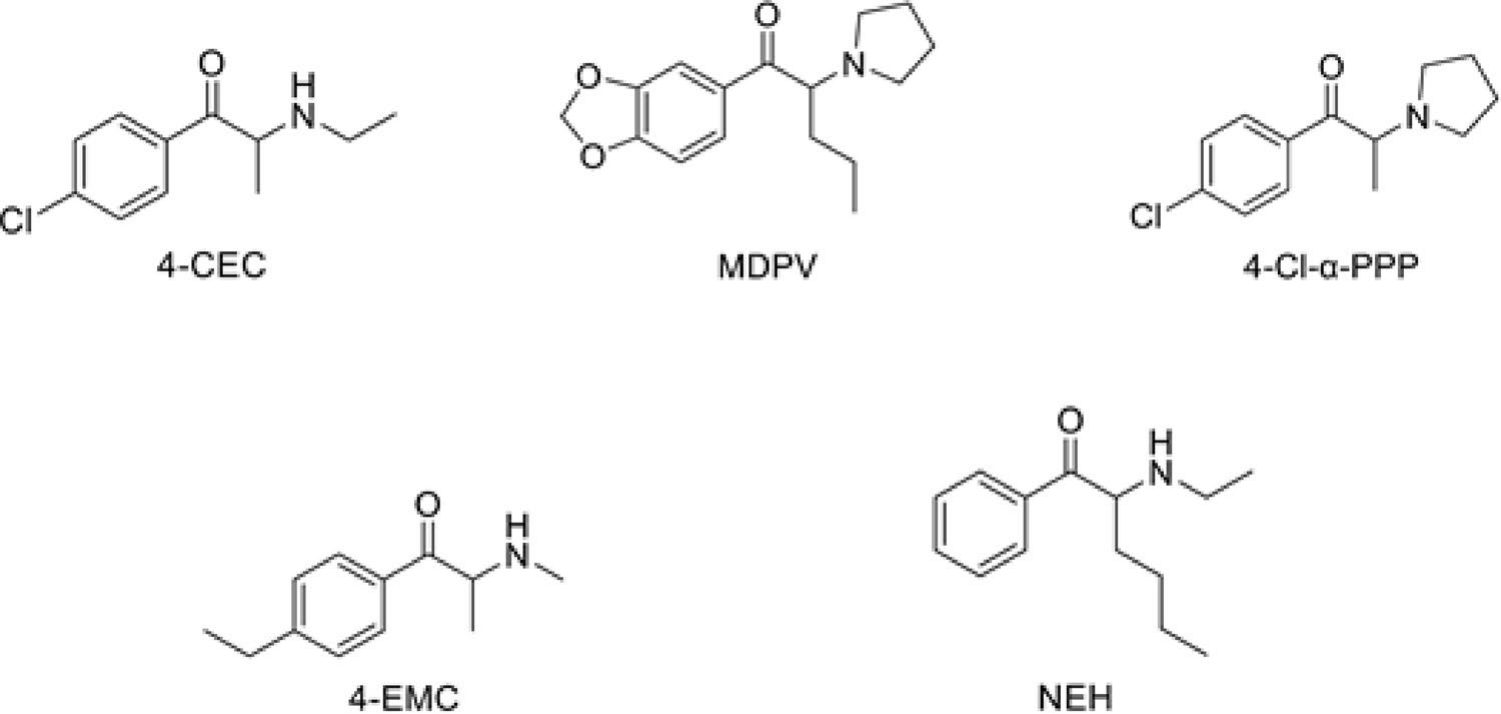
The chemical structures of 4-CEC, MDPV, 4-Cl-α-PPP, 4-EMC, and NEH

The purpose of this study was to investigate the stability of five synthetic cathinones: 4-chloroethcathinone (4-CEC), 4-ethylmethcathinone (4-EMC), N-ethylhexedrone (NEH), methylenedioxypyrovalerone (MDPV) and 4-chloro-α-pyrrolidinopropiophenone (4-Cl-α-PPP) (Fig. 4), four of which have been shown to be unstable by our research group. Given that stability in DMS is analyte-dependent (Ambach et al. 2014; Rossi et al. 2025), and that synthetic cathinone stability is highly pH-dependent (Aldubayyan et al. 2021), this study aimed to disentangle the influence of DMS from pH by investigating the stability of these compounds in liquid urine and dried urine spots (DUS) across controlled acidic and mildly basic pH conditions. Conditions from our research group’s previous study by Aldubayyan et al. (2024) (Aldubayyan et al. 2024) have been replicated for a total study length of 14 days at room temperature (25 °C) and refrigerated at 4 °C. Furthermore, stability samples were analysed by VeriSpray Paper Spray Ionisation to complement the speed and ease of use of DUS.

## Materials and methods

### Standards and chemicals

4-CEC, 4-EMC, NEH, MDPV and MDPV-d8 (1 mg/mL as HCl salt solutions) were purchased from Chiron (Woking, UK), 4-Cl-α-PPP (1 mg/mL) was donated from TICTAC Communications (London, UK) and mephedrone-d3 (1 mg/mL HCl salt) was purchased from Sigma-Aldrich (Gillingham, UK). LC–MS grade methanol (MeOH), acetonitrile (ACN) and formic acid were purchased from Fisher Scientific (Loughborough, UK). Ultrapure water (18.2 MΩ) was used throughout from an ELGA PureLab® Chorus 1 water purification system. Spiking solutions of the synthetic cathinones were prepared by diluting the solutions (1 mg/mL) with MeOH to produce spiking solutions (20 µg/mL) and stored at −40 °C until required.

### Paper spray-tandem mass spectrometry conditions

Samples were analysed using a Thermo Fisher Scientific VeriSpray Paper Spray Ion Source coupled to a Thermo Fisher Scientific TSQ Altis Plus Triple Quadrupole (QqQ) mass spectrometer. Each sample plate contained 24 triangular-shaped Whatman ET31 chromatography paper strips, with the paper tip positioned 5 mm away from the mass spectrometry inlet during sample analysis. A 90 / 10 / 0.1 (v/v/v) composition of ACN / H_2_O / formic acid was used for both the rewet and spray solvents. For sample analysis, a total of 110 µL of the solvents were dispensed onto the paper strip in the following sequence: 10 µL with a 3 s delay, then 10 µL/s for 4 s, 10 µL every 3 s for 9 s, then 10 µL every 5 s for 15 s. A high voltage of 4.5 kV was applied to the back of the paper strip via a metal rivet to induce electrospray. All analytes were analysed in positive ionisation mode using selected reaction monitoring (SRM), with four transitions being monitored per analyte (Table S1, Supplementary material). The total run time of the method was 2 min per sample. Mass spectrometric conditions included a resolution of Q1 and Q3 at 0.7 Da FWHM, an ion transfer temperature tube of 325 °C and a CID gas pressure of 1.5 mTorr. Sequence set-up and acquisition were conducted using Thermo Fisher XCalibur™ 4.5 software, with subsequent data analysis performed using Thermo Fisher TraceFinder™ Forensic 5.1 for automated ion ratio evaluation and report generation. Chronogram peak areas were integrated automatically using a fixed detection window corresponding to the duration of sample analysis. The resulting area under the curve was used for quantitation as the analyte to internal standard (ISTD) peak area ratio.

### Sample preparation

Human drug-free urine samples were obtained from 10 donors based on written informed consent approved by the King’s College London Research Ethics Committee (RESCM-22/23-10507). All donations were pooled together and the pH measured (5.98). To assess the contribution of matrix pH, half of the pooled urine was adjusted to pH 7.63 (± 0.1) using 2 M sodium phosphate buffer to reflect the basic conditions used in the previous study. Pooled acidic and basic urine (45 µL) was aliquoted into Starlab 0.5 mL TubeOne® Microcentrifuge Tubes and spiked with 5 µL of a mixture of the synthetic cathinone solutions and ISTDs to give final concentrations of 800 ng/mL and 500 ng/mL, respectively. Mephedrone-d3 was used as the ISTD for all analytes except MDPV, which used MDPV-d8. 800 ng/mL is a toxicologically relevant average of concentrations reported for the synthetic cathinones involved in the study, as reported in Table S2 of the Supplementary material. After fortification, each sample was vortexed for 10 s. For DUS, 5 µL of fortified urine at each pH was spotted in triplicate onto individual paper strips of VeriSpray sample plates. The spotting position was standardised using the integrated plate guide to ensure uniform central deposition. The spots were left to dry in ambient laboratory conditions for 15 min. For room temperature analyses, sample plates were placed into the VeriSpray plate loader for storage, whereas for 4 °C analyses, sample plates were placed into a plastic ziplock pocket with desiccant sachets before being placed into the refrigerator. For liquid samples, fortified urine (5 µL) at each pH was spotted in triplicate onto paper strips of VeriSpray sample plates and dried as above for immediate analysis. Remaining fortified liquid urine was stored at room temperature or at 4 °C; aliquots (5 µL) were spotted in triplicate and dried on the day of subsequent analyses.

### Stability study design

For unbuffered, acidic urine, room temperature stability at approximately 25 °C was analysed short-term for up to 24 h in the following increments: 1, 2, 4, 8, 12, and 24 h. Long-term studies at room temperature and 4 °C assessed stability for up to 14 days in the following increments: 1, 2, 3, 7, and 14 days. For buffered, basic urine, stability was assessed at room temperature and 4 °C after 3 days. On the whole, samples stored under both conditions had minimal light exposure. In ambient laboratory conditions, samples were stored in a closed, light-impermeable container. The VeriSpray plate loader operates at room temperature and possesses a heavily tinted door; therefore, samples receive minimal light. Refrigerated samples were stored in the dark except when being retrieved for analysis.

The average concentration at the beginning of the study (T_0_) was recorded in ng/mL and normalised to 100%. To determine the stability of the analytes, analyte concentrations were quantified against a freshly prepared calibration curve on each day of analysis, expressed as a percentage and compared with the 100% value at T_0_. Analytes were classed as unstable once their average concentration deviated by ± 20% of T_0_ concentrations. Precision (% CV) was calculated from the triplicate samples, with an acceptability criterion of 20%. One-way analysis of variance (ANOVA) Single Factor was used to determine whether significant differences existed between conditions (pH, matrix format, and storage temperature) for each analyte (Microsoft Excel, Version 16.101.2). Pairwise comparisons between groups were subsequently conducted using two-sample t-tests assuming equal variances. To control for type I error associated with multiple testing, a Bonferroni correction was applied to the significance threshold. For six pairwise comparisons per analyte and storage condition, the adjusted threshold for significance was *p* < 0.0083 (0.05 ÷ 6). Values below this threshold were interpreted as statistically significant.

### Method validation

The method was evaluated in accordance with ANSI/ASB 036 guidelines (AAFS 2019). A seven-point calibration curve was constructed with 1/y^2^ weighting at the following concentrations: 50, 100, 200, 300, 500, 750, and 1,000 ng/mL, and regression statistics were evaluated using LINEST functions and standard error plots (Microsoft Excel). Limits of detection (LODs) were established experimentally across five calibration curve runs. Bias and precision (% CV) were calculated from three quality control concentrations at 150, 475 and 800 ng/mL throughout five runs; within-run and between-run precision as well as bias had acceptability criteria of 20%, 20% and ± 20%, respectively. Carryover was assessed by running a blank urine sample in triplicate after the largest calibrator. The average blank signal for drug-free urine was assessed by analysing one blank spot from each of the 10 individual donors and averaging the resulting chronogram areas. Interference was assessed by observing the analyte signal when a solution containing 81 commonly abused drugs, metabolites, and pharmaceuticals (Table S3, supplementary material) was processed. Furthermore, blank urine, urine fortified with analytes only, and urine fortified with ISTDs only were analysed to assess interference. The method was classed as free from interference when the analyte signal at each interference assessment was less than that of the lower limit of quantification (LLOQ) and/or ion ratios were not consistent with the target analyte. Matrix effects were evaluated based on guidelines by Matusewski et al*.* (2003) whereby chronogram areas of five calibration curves of fortified urine were compared with chronogram areas of five calibration curves of fortified methanol.

## Results

### Method validation

The regression of the calibration model exhibited strong linearity across all analytes, with the coefficient of determination (R^2^) values exceeding 0.99, large F-statistics, and small residual errors indicating robust model precision and reliable coefficient estimates. Bias was between −9 and 10% across all analytes, while within-run and between-run % CV were within 19.4% and 13.8% respectively. No carryover was observed after the largest calibrator. Despite the blank urine signal for 4-EMC reaching 33.5% of the LLOQ signal, inconsistent ion ratios ruled out any true carryover. ME % were significant in the form of ion suppression, ranging from −42 to 71%, yet there was no adverse impact on analytical performance. All analytes showed bias and precision within ± 20%, indicating that ion suppression did not negatively impact the quantification of synthetic cathinones in this study. Analyte-dependent LODs ranged from 7.6 to 32.6 ng/mL, with 50 ng/mL set as the LLOQ of all analytes to ensure uniform sensitivity.

### Stability study

A summary of the stability data for each stability assessment in acidic urine is provided in Table 1. At room temperature, short-term assessments of up to 24 h at 25 °C showed that all analytes remained stable, retaining 91–109% of their initial concentrations. Across longer-term studies, all analytes retained stability for 48 h; beyond this, 4-CEC and NEH gradually but steadily degraded, eventually leading to instability of NEH by day 3 with 71% of the initial concentration remaining, and instability of 4-CEC by day 7 with 69% remaining. NEH continued to degrade until day 14, where a final 41% of the original concentration was retained. Similarly, 4-CEC retained 56% by the conclusion of the study. Conversely, 4-Cl-α-PPP, MDPV, and 4-EMC maintained stability for the entire 14 day period, with 101%, 96%, and 88% respectively, of the initial concentration remaining. In refrigerated conditions (4 °C), all analytes except NEH remained stable for the entire 14-day period, with concentrations ranging from 91 to 110% of their original values. NEH became unstable by day 7, unusually displaying a concentration increase to over 150% before decreasing slightly to 130% by day 14. In summary, four of the five synthetic cathinones remained stable at room temperature for up to three days, declining to three remaining stable for the remainder of the 14 days. At 4 °C, four of the five synthetic cathinones remained stable for 14 days. NEH displayed more degradation than the other analytes; interestingly, the degradation profile of NEH remained consistent across matrix types at room temperature, yet showed anomalous concentration increases under colder storage conditions in DUS. These results initially appeared to contrast with Aldubayyan et al. (2024), where all analytes except MDPV displayed significant degradation in mildly basic urine (pH 7.63) within three days at room temperature (22–23 °C) and 14 days at 4 °C (Aldubayyan et al. 2024). To clarify this discrepancy, additional experiments were performed under matched pH conditions.

**Table 1:** Observed stability of synthetic cathinones in DUS under acidic conditions at room temperature and 4 ºC

Analyte	Storage condition	Analyte remaining 1 h		Analyte remaining 2 h		Analyte remaining 4 h		Analyte remaining 8 h		Analyte remaining 12 h		Analyte remaining Day 1		Analyte remaining Day 2		Analyte remaining Day 3		Analyte remaining Day 7		Analyte remaining Day 14	
		Mean (%)	% CV	Mean (%)	% CV	Mean (%)	% CV	Mean (%)	% CV	Mean (%)	% CV	Mean (%)	% CV	Mean (%)	% CV	Mean (%)	% CV	Mean (%)	% CV	Mean (%)	% CV
4-CEC	Room temp	97	6	105	5	93	5	99	2	95	4	91	6	85	0	82	5	69	1	56	2
	4 °C	-	-	-	-	-	-	-	-	-	-	102	6	95	4	101	4	102	2	99	3
4-Cl-α-PPP	Room temp	97	7	100	13	89	1	103	4	95	8	109	7	104	1	106	2	99	5	101	1
	4 °C	-	-	-	-	-	-	-	-	-	-	116	9	107	4	110	6	99	3	91	1
4-EMC	Room temp	103	3	110	3	105	4	108	1	107	2	102	2	100	3	91	4	94	1	88	1
	4 °C	-	-	-	-	-	-	-	-	-	-	102	3	101	4	98	5	114	1	110	1
MDPV	Room temp	100	2	97	5	101	1	95	1	98	2	94	4	95	1	96	0	95	0	96	0
	4 °C	-	-	-	-	-	-	-	-	-	-	96	4	98	1	98	1	99	0	99	0
NEH	Room temp	112	0	116	4	105	2	107	3	106	2	102	1	82	4	71	2	60	1	41	7
	4 °C	-	-	-	-	-	-	-	-	-	-	117	4	110	8	107	7	155	4	130	9

“-” indicates not assessed; 1–12 h stability assessments were performed at room temperature only. Reported stability values represent relative stability, calculated from analyte to ISTD chronogram area ratios normalised to T_0_

To address the impact of urinary pH alongside the influence of matrix form, unmodified urine at pH 5.98 and buffered urine adjusted to pH 7.63 were assessed in liquid urine and DUS at room temperature and 4 °C for up to 3 days. The stability of synthetic cathinones in liquid urine and DUS at room temperature under acidic and mildly basic conditions is summarised in Table 2, with pairwise t-test results provided in Supplementary Material (Table S4). A Bonferroni-corrected significance threshold of *p* < 0.0083 was applied for all pairwise comparisons. Under acidic conditions, most analytes were stable at day 3 in both liquid and dried urine. For example, 4-Cl-α-PPP (102% liquid vs 106% DUS), 4-EMC (100% liquid vs 91% DUS), and MDPV (99% liquid vs 96% DUS) showed no significant difference between liquid and dried urine (*p* = 0.019, *p* = 0.140, and *p* = 0.021, respectively). Conversely, 4-CEC (71% liquid vs. 82% DUS) and NEH (115% liquid vs. 71% DUS) displayed significant differences between liquid urine and DUS (*p* < 0.0083), but with a marked difference in stability kinetics. 4-CEC remained stable when stored as DUS, however, NEH performed better when stored as liquid urine. Acidic urine preserved stability more effectively compared to basic urine, which displayed highly variable recoveries. 4-CEC degraded almost completely in basic urine (3% liquid vs 10% DUS) with very significant differences between the matrix forms (*p* = 3.3 × 10^–9^ and *p* = 2 × 10^–8^, respectively), in agreement with acidic results that showed 4-CEC benefited from being stored as DUS. NEH and 4-Cl-α-PPP showed anomalously large over-recovery in liquid urine compared to that of DUS (190% vs 14% and 716% vs 157% respectively); 4-EMC also degraded in basic urine (153% liquid vs 62% DUS) within the observation period. Although the two matrix forms are statistically significantly different in all three analytes (*p* < 0.0083), the pronounced instability of NEH, 4-Cl-α-PPP and 4-EMC in basic urine limits the relevance of this comparison. MDPV was the only analyte remaining stable across both pH conditions and matrix forms (between 91 and 96%), with no significant difference displayed except between acidic and basic liquid urine (*p* = 0.0022).

**Table 2:** Observed stability of synthetic cathinones in liquid urine and DUS under acidic and basic conditions at room temperature (n = 3)

Analyte	pH	Matrix	Analyte remaining Day 3	
			Mean (%)	% CV
4-CEC	Acidic	Liquid urine	71	1
		DUS	82	5
	Basic	Liquid urine	3	4
		DUS	10	4
4-Cl-α-PPP	Acidic	Liquid urine	102	4
		DUS	106	2
	Basic	Liquid urine	716	5
		DUS	157	5
4-EMC	Acidic	Liquid urine	100	2
		DUS	91	4
	Basic	Liquid urine	153	17
		DUS	62	3
MDPV	Acidic	Liquid urine	99	1
		DUS	96	0
	Basic	Liquid urine	91	5
		DUS	96	1
NEH	Acidic	Liquid urine	115	9
		DUS	71	2
	Basic	Liquid urine	190	12
		DUS	14	9

Reported stability values represent relative stability, calculated from analyte to ISTD chronogram area ratios normalised to T_0_

At 4 °C, the stability of synthetic cathinones in liquid urine and DUS under acidic and basic conditions is shown in Table 3, with pairwise t-test results provided in Supplementary Material (Table S5). Under refrigerated conditions, acidic urine continued to support stability, with all analytes remaining stable in liquid urine and as DUS, ranging between 95 to 115% and 98 to 110% respectively. There was no statistical significance for the analytes in acidic urine between the two matrix forms except for 4-EMC, whereby recoveries were 98% in DUS and 105% in liquid urine (*p* = 0.0027). Comparisons with basic urine again highlighted pH as the key determinant in the study. At day 3, 4-CEC concentrations were reduced in both formats (43% liquid vs. 60% DUS); as with room temperature assessments, despite statistically significant differences between liquid urine and DUS, the overall instability of 4-CEC in basic urine limits the relevance of this observation. According to the ± 20% stability criterion, 4-Cl-α-PPP was marginally unstable in both matrices (121% liquid vs 120% DUS) with no statistically significant difference observed between them. 4-EMC and MDPV remained stable at 101% liquid vs 92% DUS and 94% liquid vs 92% DUS, however, only 4-EMC demonstrated a significant difference in stability (*p* = 0.0058) between liquid urine and DUS. NEH showed a significant difference in stability dependent on the matrix format, with 106% recovery in liquid urine compared to 58% in DUS, in agreement with observations for NEH in acidic urine at room temperature, whereby the analyte benefited from storage in liquid urine over DUS.

**Table 3:** Observed stability of synthetic cathinones in liquid urine and DUS under acidic and basic conditions at 4ºC (n = 3)

Analyte	pH	Matrix	Analyte remaining Day 3	
			Mean (%)	% CV
4-CEC	Acidic	Liquid urine	95	2
		DUS	101	4
	Basic	Liquid urine	43	2
		DUS	60	2
4-Cl-α-PPP	Acidic	Liquid urine	101	3
		DUS	110	6
	Basic	Liquid urine	121	4
		DUS	120	5
4-EMC	Acidic	Liquid urine	105	2
		DUS	98	5
	Basic	Liquid urine	101	4
		DUS	92	2
MDPV	Acidic	Liquid urine	100	1
		DUS	98	1
	Basic	Liquid urine	94	1
		DUS	92	1
NEH	Acidic	Liquid urine	115	6
		DUS	107	7
	Basic	Liquid urine	106	2
		DUS	58	10

Reported stability values represent relative stability, calculated from analyte to ISTD chronogram area ratios normalised to T_0_

Across all experiments, integrated recovery data and t-test assessments demonstrate that acidic urine consistently provided a protective effect on synthetic cathinone stability, with few isolated format differences. By contrast, basic urine was associated with rapid degradation or anomalous over-recoveries with more statistically significant differences in matrix format for nearly all analytes. Acidic matrices also preserved analytes for up to 14 days, while basic matrices caused significant degradation within three days for the majority of analytes.

## Discussion

This study aimed to investigate the stability of the five synthetic cathinones in both liquid and DUS in acidic and mildly basic pH conditions across 14 days to determine which factor can aid in improving analyte stability. Stability assessments below 4 °C were deliberately omitted, as prior data from Aldubayyan et al. (2024) confirm that the analytes remain stable at −20 °C for up to 30 days even at basic pH (Aldubayyan et al. 2024), making further investigations into improved stability at colder temperatures within 14 days redundant. The time points were selected based on the complete instability of the analytes within 14 days in liquid urine; extending the stability assessment beyond this window was therefore unnecessary. Conversely, the absence of short-term time points in Aldubayyan’s study prompted the inclusion of hourly assessments in this investigation to capture early degradation kinetics and provide additional insight into the stability dynamics of synthetic cathinones. Similarly, assessments in basic urine were limited to three days as the experiment design focused on a target comparison to clarify the influence of pH, not replicate the full 14 day time course; furthermore, prior results had already demonstrated complete instability after three days under basic urinary conditions (Aldubayyan et al. 2024). The analytes selected were based on the degradation profiles of highly unstable analytes reported by Aldubayyan et al. (2024) (Aldubayyan et al. 2024), enabling a thorough investigation into improved stability profiles in DUS. An exception was MDPV, which generally exhibited exceptional stability in liquid urine, but was included in the current study as a positive control and to confirm previously reported stability data for this analogue (Adamowicz and Malczyk 2019; Ciallella et al. 2020; Glicksberg and Kerrigan 2018; Aldubayyan et al. 2024).

To ensure consistency across all conditions, both analytes and ISTDs were spiked at T_0_ for every sample, including liquid urine and DUS. This approach avoided introducing a secondary variable related to the timing of ISTD addition, which would have hindered direct comparison between matrices. As mephedrone-d3 was used as a surrogate deuterated synthetic cathinone, it may also undergo degradation. As a result, the stability values reported here reflect relative stability using analyte to ISTD ratios, rather than absolute signal loss. This does not affect the comparative outcomes of the study, as all conditions were treated identically, but it should be recognised as an inherent feature of the study design.

The most significant finding of this study was the confirmation that urinary pH is the dominant factor in controlling analyte stability, with almost all analytes performing better in acidic urine regardless of the storage temperature, while matrix format has varying effects. The influence of urinary pH on synthetic cathinone degradation pathways is well-documented. Extensive studies have shown that samples with an acidic pH (~ 4) exhibit considerably improved stability of various synthetic cathinone analogues compared with samples with a basic pH (~ 8) (Glicksberg and Kerrigan 2018; Adamowicz and Malczyk 2019; Tsujikawa et al. 2012; Glicksberg et al. 2018), emphasising a somewhat protective effect of an acidic matrix, and enabling urinary pH to be a strong predictor of synthetic cathinone decay. Many synthetic cathinones are primary or secondary amines; therefore, at basic pH, synthetic cathinones are more likely to undergo base-catalysed hydrolysis or oxidative deamination. Urinary acidification could suppress these pathways by limiting the formation of deprotonated, reactive species and microorganism growth that can produce basic by-products (Zaitsu et al. 2008). However, the mildly acidic (pH 5.98) urine in this study is unlikely to have fully suppressed microbial proliferation, as growth has persisted at pH 5–8 (Erdogan-Yildirim et al. 2011). The improved stability observed here therefore likely reflects suppression of base-catalysed degradation rather than total microbial inhibition.

Notably, 4-Cl-α-PPP, 4-CEC and NEH exhibited distinct degradation kinetics in basic urine. 4-CEC showed remarkable instability in basic urine in both storage conditions, potentially due to its simple secondary amine and halogenated ring substitution, which offer minimal steric protection. These structural features make 4-CEC susceptible to base-catalysed degradation as mentioned previously, while the activated ring further facilitates instability. Similarly, the extreme over-recovery (> 700%) of 4-Cl-α-PPP is likely an artefact arising from hydrolysis or deamination, which in turn may promote the formation of adducts, thereby enhancing signal intensity. Regardless, in both cases, DUS did offer some improved degradation kinetics, aligning with previous reports suggesting that DMS can attenuate degradation processes through matrix desiccation and reduced enzymatic activity (Jacques et al. 2022). By contrast, varied sample recoveries of NEH point to unique analyte-paper interactions. For this analyte, DUS in fact reduces stability in basic urine, which could be due to the use of a hydrophilic, cellulose-based paper substrate. Despite the long alkyl chain of NEH increasing its overall lipophilicity, cellulose-based paper may instead simply retain the polar functional groups leaving them exposed to structural degradation pathways.

Structural features of synthetic cathinone analogues play a central role in determining their stability. Analogues containing 3,4-methylenedioxy ring substitutions and N-pyrrolidine substitutions, such as MDPV, have been shown to exhibit significantly less degradation than synthetic cathinone compounds without these features, complementing the findings in this study. It has been speculated that the combination of these two structural features has a dual-stabilising effect (Ellefsen et al. 2016; Glicksberg and Kerrigan 2018). Analogues containing tertiary amines, such as NEH, have been found to exhibit greater stability than secondary amines (Glicksberg et al. 2018), such as 4-EMC, followed by structures containing secondary amines with or without halogenated ring substitutions, such as 4-Cl-α-PPP and 4-CEC, being the most unstable (Adamowicz and Malczyk 2019; Aldubayyan et al. 2021; Glicksberg and Kerrigan 2017). These observations are largely reflected in the findings in this study, whereby 4-CEC was found to be unstable in acidic urine within seven days at room temperature; however, the instability of NEH is contradictory to the previously documented findings relating to its structure, exhibiting the most pronounced degradation kinetics across the study at room temperature in both pH conditions and matrix forms except acidic liquid urine, and a sudden loss of stability in basic DUS at 4 ºC only. As previously highlighted, this may be due to specific interactions between the analyte, DUS, and the paper substrate.

From a forensic toxicology standpoint, these results have several implications. Firstly, the use of urine in a forensic context is not obsolete, despite whole blood often being the matrix of choice, with case reports of fatal and non-fatal concentrations of synthetic cathinones in urine routinely reported (Froberg et al. 2015; Wright et al. 2013; Pieprzyca et al. 2023, 2022; Kuropka et al. 2023). Secondly, the use of DUS still presents considerable toxicological impact. The approach demonstrated robustness across variable urine matrices and reduced-dependence on cold-chain storage. Although spot homogeneity was not separately assessed, potential spatial variation within DMS is unlikely to influence results, as PSI utilises the entire wetted area for ionisation rather than discrete punches, reducing position-related bias. This suggests that one could deposit urine on to a paper strip for transport, storage, or in the case of sample backload, for up to 48 h to maintain analyte integrity, whether for subsequent PSI analyses or traditional LC–MS techniques.

Future considerations in synthetic cathinone stability studies include investigations into their photostability, and pH monitoring over the course of a study. Although light exposure was controlled in the present study, with samples stored in the absence of direct light, future studies could examine photodegradation as an independent variable. Cathinone within the *Catha edulis* plant has been found to be converted into dimers or inactive metabolites under natural sunlight (Katz et al. 2014), likely due to its β-keto-phenethylamine structure, which is conserved across many synthetic cathinones. Therefore, there is a mechanistic basis to hypothesise that synthetic cathinone analogues possess features conducive to similar degradation pathways. Given that these compounds share photochemically active functional groups, such as the β-keto group and aromatic ring, it is reasonable to infer that synthetic cathinones may also be prone to light-induced degradation under environmental or analytical conditions. This possibility is further supported by the general instability of synthetic cathinones and their tendency to degrade under various stressors, though photodegradation has yet to be specifically isolated and quantified in a controlled experimental framework. Additionally, dynamic changes in urinary pH can arise from the bacterial decomposition of amino acids and urea, leading to the formation of amines and ammonia, and therefore the alkalisation of urine (Zaitsu et al. 2008). Such fluctuations in pH may influence the stability of some analytes over time, confounding interpretation of the results. Due to the nature of DMS, there is an inability to monitor pH throughout associated studies, however, it is important to be cognisant of potential pH fluctuations when assessing liquid urine. Another avenue of future work could incorporate external calibration or delayed ISTD addition to aid in distinguishing absolute from relative degradation kinetics.

## Conclusion

This study uniquely contributes to the lack of explicit mechanistic experiments investigating the protective effect of acidic pH in combination with addressing common storage and transportation dilemmas encountered with traditional liquid sampling. In summary, synthetic cathinone stability in urine is governed primarily by matrix pH, not whether the urine is dried or liquid. While DUS rarely offered an intrinsic stability advantage, acidic conditions markedly improved analyte stability compared with basic conditions. These results reconcile previous observations of instability and underscore the importance of accounting for pH in biological stability studies. The application of forensically relevant urine samples to cellulose-based chromatography paper highlights the potential benefits of a DUS approach for forensic toxicology, regardless of subsequent coupling to PSI techniques, especially where fast and resource-efficient handling is required. This effect is particularly valuable under room temperature conditions, where the avoidance of cold storage not only reduces logistical constraints but also lowers costs. Notably, simple benchtop storage with appropriate shielding from light and environmental contaminants may suffice to maintain analyte integrity, reinforcing the practicality of this method in operational settings where chain-of-custody has to be evidenced.

Future work should focus on standardising experimental protocols and further elucidating additional mechanisms, such as photostability, urinary pH monitoring, ISTD addition, and degradation pathways that may contribute to the stability kinetics of synthetic cathinones in urine. This will be essential to develop robust, widely applicable guidelines for synthetic cathinone storage and analysis in forensic contexts.

## Supplementary Information

Below is the link to the electronic supplementary material.

Supplementary Material 1
