# Supplementary material for: Evaluating the stability of synthetic cathinones in liquid urine and dried urine spots: impact of pH and storage conditions
Source: Arch Toxicol. 2025 Dec 21;100(3):931–41. doi: 10.1007/s00204-025-04272-0 (PMC12967546; doi:10.1007/s00204-025-04272-0)
Supplement: Supplementary file 1 — Supplementary Material 1 [file 204_2025_4272_MOESM1_ESM.docx]

**Evaluating the Stability of Synthetic Cathinones in Liquid Urine and Dried Urine Spots: Impact of pH and Storage Conditions**

Journal name: Archives of Toxicology

*Stefania Boccuzzi^1^, David Cowan^1^, Paul I Dargan^2,3^, Edward Goucher^4^, and Vincenzo Abbate^1^*.*

^1^Department of Analytical, Environmental & Forensic Sciences, Faculty of Life Sciences & Medicine, King’s College London, London, UK

^2^Clinical Toxicology, Faculty of Life Sciences and Medicine, King's College London, London, UK

^3^Clinical Toxicology, Guy's and St Thomas' NHS Foundation Trust and King's Health Partners, London, UK

^4^Thermo Fisher Scientific, San Jose, CA, USA

*Corresponding author, vincenzo.abbate@kcl.ac.uk.

| **Table S1. Compound specific mass spectrometry settings for SRM acquisitions. Quantifier ion transitions are denoted by *** | | | | | |
| --- | --- | --- | --- | --- | --- |
| *Compound* | *Precursor (m/z)* | *Product (m/z)* | *Ion ratios (%)* | *Collision Energy (eV)* | *RF Lens (V)* |
| 4-CEC | 212.1 | *159.1 | 100 | 18 | 50 |
| 4-CEC | 212.1 | 144.1 | 74 | 28 | 50 |
| 4-CEC | 212.1 | 166.0 | 31 | 18 | 50 |
| 4-CEC | 212.1 | 131.1 | 22 | 25 | 50 |
| 4-Cl-α-PPP | 238.2 | *139.0 | 100 | 25 | 60 |
| 4-Cl-α-PPP | 238.2 | 167.0 | 72 | 18 | 60 |
| 4-Cl-α-PPP | 238.2 | 185.1 | 7 | 23 | 60 |
| 4-Cl-α-PPP | 238.2 | 103.1 | 57 | 33 | 60 |
| 4-EMC | 192.1 | *146.1 | 100 | 18 | 45 |
| 4-EMC | 192.1 | 159.1 | 20 | 19 | 45 |
| 4-EMC | 192.1 | 161.1 | 17 | 12 | 45 |
| 4-EMC | 192.1 | 131.0 | 21 | 30 | 45 |
| MDPV | 276.2 | *126.1 | 100 | 30 | 50 |
| MDPV | 276.2 | 135.0 | 87 | 30 | 50 |
| MDPV | 276.2 | 175.1 | 82 | 25 | 50 |
| MDPV | 276.2 | 205.1 | 82 | 18 | 50 |
| MDPV-d8 | 284.2 | *134.1 | 100 | 27 | 70 |
| MDPV-d8 | 284.2 | 149.0 | 42 | 35 | 70 |
| MDPV-d8 | 284.2 | 175.0 | 79 | 25 | 70 |
| MDPV-d8 | 284.2 | 205.0 | 67 | 17 | 70 |
| Mephedrone-d3 | 181.1 | *148.1 | 100 | 22 | 40 |
| Mephedrone-d3 | 181.1 | 119.1 | 21 | 22 | 40 |
| Mephedrone-d3 | 181.1 | 130.0 | 7 | 35 | 40 |
| Mephedrone-d3 | 181.1 | 145.1 | 52 | 20 | 40 |
| NEH | 220.2 | *146.1 | 100 | 18 | 50 |
| NEH | 220.2 | 130.1 | 69 | 35 | 50 |
| NEH | 220.2 | 175.1 | 28 | 15 | 50 |
| NEH | 220.2 | 118.1 | 59 | 25 | 50 |

| **Table S2. Case reports of fatal and non-fatal concentrations of MDPV, 4-CEC, and NEH found in urine. At the time of publication, there is no case report detailing 4-EMC or 4-Cl-α-PPP intoxication or fatalities.** | | | |
| --- | --- | --- | --- |
| *Drug* | *Concentration (ng/mL)* | *Reference* | *Notes* |
| MDPV | 509-3,100 | [[37](#_ENREF_37)] | Non-fatal intoxication |
|  | 670 | [[37](#_ENREF_37)] | Fatal intoxication |
|  | 760 | [[38](#_ENREF_38)] | Case no. 1, the official cause of death is diabetic ketoacidosis. MDPV concentration is not within fatal ranges, but a link between the death and MDPV use is suggested |
|  | 3,800 | [[38](#_ENREF_38)] | Case no. 2, coadministration with multiple drugs, the official cause of death was complications of HIV in a setting of MDPV abuse; MDPV concentrations are not within fatal ranges. |
| 4-CEC | 4,512 | [[40](#_ENREF_40)] | Supplementary info, table S2. Case no. 19, coadministration with alcohol at 1.3 mg/mL (blood) and 2.3 mg/mL (urine), fatal toxicity leading to death |
|  | 582 | [[40](#_ENREF_40)] | Supplementary info, table S2. Case no. 20, no coadministration, fatal toxicity leading to death |
| NEH | 60 | [[39](#_ENREF_39)] | Case no. 13, coadministration of 4-Cl-α-PVP, amphetamine, THC-COOH, and ketoprofen, fatal toxicity leading to death |
|  | 440 | [[39](#_ENREF_39)] | Case no. 14, coadministration with 4-Cl-α-PVP, ketoprofen, and alcohol, no comment on toxicity; the patient died after being assaulted |
|  | 920 | [[39](#_ENREF_39)] | Case no. 16, coadministration with α-PHP and etizolam, fatal toxicity leading to death |
|  | 600 | [[39](#_ENREF_39)] | Case no. 17, coadministration with α-PHP and 4-FBF, fatal toxicity leading to death |
|  | 174 | [[39](#_ENREF_39)] | Case no. 19, coadministration with α-PHP and 4-FBF, fatal toxicity leading to death |
|  | 147-1,477 | [[41](#_ENREF_41)] | Fatal intoxication, table 1 |
|  | 165 | [[39](#_ENREF_39)] | Non-fatal intoxication, table 1 |
| **Minimum concentration:** | **10 ng/mL** |  | |
| **Maximum concentration:** | **4,512 ng/mL** |  |  |
| **Mean:** | **1,194 ng/mL** |  | |
| **Median:** | **600 ng/mL** |  | |

| **Table S3. The compounds within the QCs used for interference studies and their respective concentrations.** | |
| --- | --- |
| *Analyte* | *Concentration (ng/mL)* |
| 3-methyl-fentanyl | 10 |
| 6-MAM | 250 |
| Adrafinil | 500 |
| AICAR | 100 |
| Altizide | 1000 |
| Amfepramone | 500 |
| Amphetaminil | 500 |
| Andarine | 10 |
| Bendroflumethiazide | 1000 |
| Benfluorex | 500 |
| Benzphetamine | 500 |
| Benzthiazide | 1000 |
| Benzylpiperazine | 500 |
| Butizide | 1000 |
| Carphedon | 500 |
| Cathine | 500 |
| Clobenzorex | 500 |
| Clomiphene | 200 |
| Cropropam. | 500 |
| Crotethamide | 500 |
| Cyclopenthiazide | 1000 |
| Cyclothiazide | 1000 |
| Dexamethasone | 600 |
| Diacetolol | 500 |
| Dimethylamphetamine | 500 |
| Dobutamine | 500 |
| Efaproxiral | 100 |
| Ephedrine | 500 |
| Epitizide | 1000 |
| Eplerenone | 1000 |
| Etamivan | 500 |
| Ethylphenylbutylamine | 500 |
| Etilamphetamine | 500 |
| Etilefrine | 500 |
| Exemestane metabolite | 200 |
| Famprofazone | 500 |
| Fenbutrazate | 500 |
| Fencamfamin | 500 |
| Fencamine | 500 |
| Fenetylline | 500 |
| Fenfluramine | 500 |
| Fenproporex | 500 |
| Flunisolide | 300 |
| Fulvestrant | 200 |
| Furfenorex | 500 |
| Heptaminol | 500 |
| Hydroxybromant. | 500 |
| Isometheptene | 500 |
| Letrozole metab. | 200 |
| MDMA | 500 |
| Mefenorex | 500 |
| Mephentermine | 500 |
| Methamphetamine | 500 |
| Methoxyphenamine | 500 |
| Methylephedrine | 500 |
| Methylhexaneamine | 500 |
| Methylphenidate | 500 |
| Metolazone | 1000 |
| Nadolol | 500 |
| Nikethamide | 500 |
| Norfenfluramine | 500 |
| Ostarine | 10 |
| Oxilofrine | 500 |
| Pemoline | 500 |
| Pentetrazol | 500 |
| Phenmetrazine | 500 |
| Phenpromethamine | 500 |
| Phentermine | 500 |
| Pholedrine | 500 |
| Piretanide | 1000 |
| p-OH amphetamine. | 500 |
| Polythiazide | 1000 |
| Prenylamine | 500 |
| Probenecid | 1000 |
| Prolintane | 500 |
| Propylhexedrine | 500 |
| Salmeterol | 100 |
| Sibutramine | 500 |
| Strychnine | 500 |
| Trichlormethiazide | 1000 |
| Tuaminoheptane | 500 |

| **Table S4. P-values from pairwise t-tests (two-sample assuming equal variances) comparing each analyte across liquid urine, DUS, and acidic and basic urinary pH at room temperature. Reported values are two-tailed P(T≤t). Statistically significant observations (p < 0.0083, Bonferroni-adjusted) are shown in bold.** | | | | | |
| --- | --- | --- | --- | --- | --- |
|  | 4-CEC | 4-Cl-α-PPP | 4-EMC | MDPV | NEH |
| Acidic - Liquid vs. Acidic - DUS | **4.47×10^-4^** | 1.87×10^-2^ | 1.40×10^-1^ | 2.13×10^-2^ | **2.31×10^-3^** |
| Acidic - Liquid vs Basic - Liquid | **3.33×10^-9^** | **3.99×10^-5^** | 9.21×10^-2^ | **2.23×10^-3^** | **4.25×10^-3^** |
| Acidic - Liquid vs Basic - DUS | **1.96×10^-8^** | **3.04×10^-4^** | **2.84×10^-5^** | 2.54×10^-2^ | **1.68×10^-5^** |
| Acidic - DUS vs Basic - Liquid | **2.51×10^-9^** | **3.66×10^-5^** | 1.29×10^-1^ | 9.68×10^-3^ | **6.13×10^-4^** |
| Acidic - DUS vs Basic - DUS | **1.44×10^-8^** | **8.45×10^-5^** | **9.72×10^-5^** | 5.61×10^-1^ | **2.12×10^-4^** |
| Basic - Liquid vs Basic - DUS | **4.52×10^-5^** | **5.91×10^-5^** | 1.05×10^-2^ | 2.14×10^-2^ | **6.65×10^-5^** |

| **Table S5. P-values from pairwise t-tests (two-sample assuming equal variances) comparing each analyte across liquid urine, DUS, and acidic and basic urinary pH at 4°C. Reported values are two-tailed P(T≤t). Statistically significant observations (p < 0.0083, Bonferroni-adjusted) are shown in bold.** | | | | | |
| --- | --- | --- | --- | --- | --- |
|  | 4-CEC | 4-Cl-α-PPP | 4-EMC | MDPV | NEH |
| Acidic - Liquid vs. Acidic - DUS | 4.35×10^-1^ | 1.39×10^-1^ | **2.68×10^-3^** | 1.16×10^-1^ | 7.36×10^-1^ |
| Acidic - Liquid vs Basic - Liquid | **1.66×10^-7^** | **3.72×10^-3^** | **2.56×10^-3^** | **3.77×10^-3^** | 2.99×10^-1^ |
| Acidic - Liquid vs Basic - DUS | **5.30×10^-6^** | **5.44×10^-3^** | **3.27×10^-4^** | **1.64×10^-5^** | **6.25×10^-4^** |
| Acidic - DUS vs Basic - Liquid | **9.60×10^-7^** | **3.14×10^-3^** | **8.77×10^-4^** | **3.13×10^-3^** | 6.75×10^-1^ |
| Acidic - DUS vs Basic - DUS | **1.21×10^-5^** | **5.40×10^-3^** | **3.13×10^-4^** | **2.12×10^-5^** | **2.25×10^-3^** |
| Basic - Liquid vs Basic - DUS | **5.15×10^-5^** | 4.05×10^-2^ | **5.82×10^-3^** | 1.32×10^-1^ | **1.35×10^-6^** |
